# Membrane and Electrochemical Processes for Water Desalination: A Short Perspective and the Role of Nanotechnology

**DOI:** 10.3390/membranes10100280

**Published:** 2020-10-12

**Authors:** Moon Son, Kyung Hwa Cho, Kwanho Jeong, Jongkwan Park

**Affiliations:** 1 School of Urban and Environmental Engineering, Ulsan National Institute of Science and Technology, UNIST-gil 50, Ulsan 44919, Korea; moonson619@unist.ac.kr (M.S.); khcho@unist.ac.kr (K.H.C.); 2 School of Civil, Environmental and Chemical Engineering, Changwon National University, Changwon, Gyeongsangnamdo 51140, Korea

**Keywords:** membrane process, electrochemical cell, desalination, energy efficiency, nanotechnology

## Abstract

In the past few decades, membrane-based processes have become mainstream in water desalination because of their relatively high water flux, salt rejection, and reasonable operating cost over thermal-based desalination processes. The energy consumption of the membrane process has been continuously lowered (from >10 kWh m^−3^ to ~3 kWh m^−3^) over the past decades but remains higher than the theoretical minimum value (~0.8 kWh m^−3^) for seawater desalination. Thus, the high energy consumption of membrane processes has led to the development of alternative processes, such as the electrochemical, that use relatively less energy. Decades of research have revealed that the low energy consumption of the electrochemical process is closely coupled with a relatively low extent of desalination. Recent studies indicate that electrochemical process must overcome efficiency rather than energy consumption hurdles. This short perspective aims to provide platforms to compare the energy efficiency of the representative membrane and electrochemical processes based on the working principle of each process. Future water desalination methods and the potential role of nanotechnology as an efficient tool to overcome current limitations are also discussed.

## 1. Introduction

Membrane processes have accelerated industrial use and research on water desalination owing to relatively low energy consumption (~4 kWh m^−3^) compared to previously used multiple-effect distillation and multi-stage flash thermo-based processes (>10 kWh m^−3^) [1,2]. Thus, membrane processes, such as reverse osmosis (RO), are the gold standard for water desalination [3,4,5]. In the last few decades, the energy used for seawater desalination using the RO process has decreased significantly, from ~10 kWh m^−3^ to 4 kWh m^−3^ [3,6] (~3 kWh m^−3^ in recently established plants [7]) because of the development of high-performance membranes and energy recovery devices and because the RO process does not require thermo-energy to operate [1]. This RO energy consumption implies a thermodynamic energy efficiency of approximately 20% when considering the theoretical minimum energy consumption of ~0.8 kWh m^−3^ for seawater desalination [2]. However, the ~4 kWh m^−3^ level has not been drastically reduced, despite further technological advances because of inevitable membrane resistance and friction loss—inherent limitations of the RO process [8,9].

The success of the electrochemical process for desalination depends on the relatively low energy consumption compared to that of the membrane process [10]. The working principle of the electrochemical process is mostly attributed to salt adsorption by electrodes or ion migration by ion-exchange membranes in the system [11,12,13]. Thus, the relatively limited salt adsorption (or removal) or the capacity of the electrochemical processes often limits the application of electrochemical processes to brackish water desalination [10,13]. The limited ion removal capacity of the electrochemical process implies that the overall productivity and energy efficiency (according to the extent of desalination) and the energy consumption of the electrochemical and membrane processes should be compared [14,15]. A recent study indicates that capacitive deionization (CDI), one of the most widely studied electrochemical desalination technologies, uses less energy than RO but has a lower energy efficiency due to lower productivity and a lower extent of desalination [16]. Therefore, an understanding of the process evaluation matrix (i.e., thermodynamic energy efficiency versus overall productivity) is needed to understand the direction of future desalination research.

From this perspective, a brief introduction of the membrane processes, the most widely employed desalination process, and its working principle are presented first. Subsequently, several important aspects of currently studied electrochemical cells are described. Because detailed research or review articles for each desalination process have been published recently [14,15,16,17,18,19], this perspective attempts to introduce several major findings from each study. A performance evaluation matrix of theoretical (or practical) energy consumption with an emphasis on thermodynamic energy efficiency is introduced based on the characteristics of the representative membrane (RO) and electrochemical (CDI) processes. Additionally, the applications of these processes for recovering energy and valuable resources are briefly presented. Finally, the future direction of water desalination technologies and the possible contribution of nanotechnology are discussed as efficient tools to overcome current limitations.

## 2. Membrane Process

### 2.1. Reverse Osmosis

**Figure 1:**
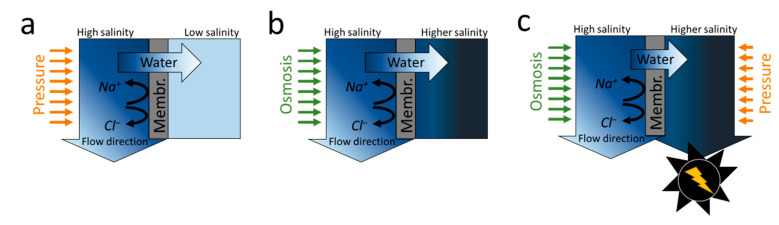
Schematic and working principle of (**a**) pressure-driven (RO) or (**b**) osmotic-driven (FO) membrane processes. (**c**) Membrane-based renewable energy production process (PRO) using a turbine (black) driven by pressurized water flow.

The RO process utilizes a hydraulic pressure higher than the osmotic pressure across a semi-permeable polymer membrane, such as thin-film composite (TFC) membranes (Figure 1a). Controlling the concentration polarization (CP) of ions in the RO process is particularly important, as CP increases the osmotic pressure on the membrane surface, thereby requiring a driving pressure higher than it practically needs. The use of cross-flow filtration, in which the feed solution flows along the membrane surface, can reduce the CP of the ions in the feed side boundary layer, resulting in high energy efficiency (>20%; <4 kWh m^−3^) [3,20,21]. The development of a superior low resistance TFC RO membrane with high selectivity and permeability has greatly improved the RO process’ energy efficiency, thereby reducing the energy consumption in commercial RO plants from >10 kWh m^−3^ to <4 kWh m^−3^ [3,6]. In past decades, most research has focused on improving the TFC RO membranes’ performance from ~1 LMH/bar to 5.77 LMH/bar without sacrificing the salt rejection (Table 1) [22,23,24,25]. Nevertheless, the application of highly permeable membranes to commercial-sized existing modules could increase the extent of CP within the module and accordingly makes process optimization difficult [19,26]. Thus, developing a new module with shorter vessel length could take advantage of a highly permeable membrane. Membrane fouling should also be considered when a highly permeable membrane is employed as the increased amount of water filtered could increase the fouling propensity of the membrane. However, the fouling test for the highly permeable membrane has been tested with only a few foulants [23,25], and the evaluation of chlorine resistance of the membrane, which is required for chlorine-based disinfection to mitigate biofouling, has not been sufficiently performed. For these reasons, current research has focused mainly on increasing selectivity and reducing membrane fouling instead of permeability. The desire to develop ideal desalination membranes has furthered the development of channel-type membranes (aquaporin, protein, carbon nanotube, and nanofiber) or ultra-thin two-dimensional materials [25,27,28,29,30,31]. However, the extent to which the new membranes increase the energy efficiency of RO is poorly understood, because most membranes are in the early stages of development and remain uncommercialized.

Two major factors used to evaluate the RO process, water flux (*J_w_;* L m^−2^ h^−1^), and salt rejection (*R*; often assumed to be >99%), can be calculated as follows:

(1) $$J_{w}=A\times P=\frac{\Delta V_{w}}{A_{m}\Delta t}$$

(2) $$R=\left(1-\frac{C_{p}}{C_{f}}\right)\times 100\left(\%\right)$$

where *A* is membrane permeance (often assumed to be >1.2 L m^−2^ h^−1^ bar^−1^), ∆*V_w_* is the decreased volume of water on the feed side (L), *A_m_* is the effective membrane area (m^−2^), Δ*t* is permeation time (h), and *P* is applied pressure (bar). *C_p_* and *C_f_* are the permeated and feed water concentrations, respectively.

The Gibbs free energy of separation *(*∆*g*; kWh m^−3^) and the specific energy consumption (*SEC*) are calculated to obtain the thermodynamic energy efficiency (*TEE*), the universal factor to compare each process.

(3) $$TEE=\frac{\Delta g}{SEC}\times 100(\%)$$

The practical energy consumption of the RO process in a single-stage system can be calculated as:

(4) $$SEC_{RO}=\frac{1}{\eta_{p}}\left[\frac{\pi^{*}}{Y}\left(\frac{1}{1-Y}-\eta_{E}\right)+\frac{\Delta P_{circ}}{Y}+\Delta P_{chan}^{r=1}\right]$$

where *η_p_* is the pump efficiency (often assumed to range from 0.8–0.85), *η_E_* is the efficiency of the energy recovery device (often assumed to range from 0.90–0.98), and *Y* is the fractional water recovery (typically assumed to reach up to 0.5) [44,45,46]. $\Delta P_{circ}$ and $\Delta P_{chan}^{r=1}$ are the frictional pressure drops in the piping and membrane module, respectively [47]; π* is the osmotic pressure of the feed water at the membrane wall, which can be defined as π* = *π*_0_ · exp(*J_w_*/*k*), with the following assumptions: (1) the solute is completely rejected by the RO membrane; (2) the ratio of solute concentrations (*C_w_*/*C_b_*) is approximately equal to the ratio of osmotic pressures, π*/*π*_0_ [48]; *k* is the solute mass-transfer coefficient. Equation (4) can be used to estimate the practical energy consumption that considers the CP phenomenon on the membrane surface for a given RO membrane water flux. Equation (4) can be derived with a practical minimum *SEC* model for one-stage RO modified by applying π* to the existing model [47]. The use of *π*_0_ in the model represents the condition at the limit of the thermodynamic restriction of cross-flow RO desalting without the CP phenomenon, ∆*P* = ∆*π*_0_/(1 − *Y*) [47,49].

### 2.2. Forward Osmosis

The forward osmosis (FO) process uses osmotic pressure rather than the hydraulic pressure (chemical potential) of the solution (Figure 1b). Water molecules in the feed solution (target water) briefly migrate to the draw solution (high saline water) because of osmosis differences. The use of osmotic pressure has rendered the FO process a next-generation desalination technology with higher fouling reversibility than RO because of the lack of hydraulic pressure [50]. However, one of the most problematic issues with FO is finding a suitable draw solute [51,52,53]. In the early stages of FO research, ammonium bicarbonate (draw solute) received much attention because of its high volatility at relatively low temperatures [54]. However, a remaining technical difficulty is that any form of draw solute must have a higher chemical potential than brine and be simple to recover. Ammonium bicarbonate, considered one of the most promising draw solutes, also requires an additional post-treatment process to regenerate with low-grade heat [55]. Therefore, maximizing the chemical potential difference by creating a high CP on the draw side of an FO membrane with minimal use of draw solute could be an alternative way to commercialize the FO process. Recently, a technique for obtaining high CP using a small amount of draw solute by applying electricity to a system was developed [56], but research towards lower energy consumption should continue.

When two solutions with different salinities come in contact with a membrane (such as in the FO process), water molecules flow from the low-concentration solution (feed solution) to the high concentration solution (draw solution). Water molecules are transferred from the feed to the draw solution and drive a turbine to produce energy when the high-concentration solution is operated in a pressurized state in a process called pressure-retarded osmosis (PRO) (Figure 1c). PRO is being studied to produce renewable energy where two solutions with different salinities, such as seawater and river water, are available.

### 2.3. Hybrid Membrane Processes

Hybrid membrane processes, such as NF–RO (NF: nanofiltration), FO–RO, and RO–PRO have been developed to lower energy consumption by combining unit processes such as NF, RO, FO, and PRO [9,57,58,59,60,61,62,63]. The processes have shown the reduced energy consumption of water desalination (mostly for RO) by decreasing the salinity of the feed solution or by harvesting salinity gradient energy from RO brine that is generally doubled total ion concentrations of the seawater. However, the high installation and maintenance costs of each process remain a challenge. For example, when using seawater and river water together, the PRO process has less than half the flux of the RO process because of differences in the osmotic pressure (<12 bar for PRO at maximum power density) and hydraulic pressure (~60 bar at 50% recovery for RO) for each process [4,64,65]. Thus, according to the mass balance, the membrane surface area of the PRO process should be twice that of the RO process. This mass balance issue could be applied to other hybrid processes, such as NF–RO or FO–RO, as the optimized water flux of each process is mostly in the order of FO < RO < NF [9,66]. Therefore, capital and operating costs can be a critical issue and should be considered in the design of hybrid membrane processes.

## 3. Electrochemical Cells

### 3.1. Capacitive Deionization

**Figure 2:**
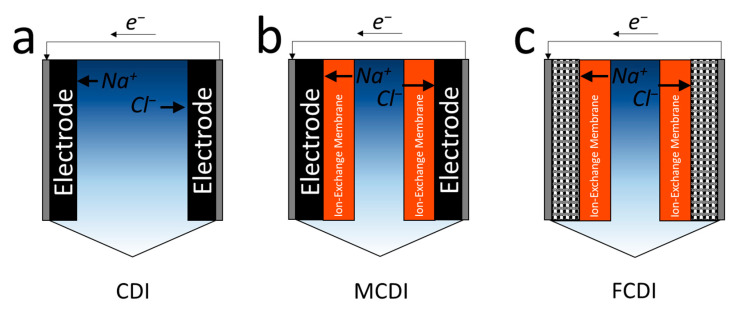
Schematic and working principle of (**a**) capacitive deionization (CDI), (**b**) membrane capacitive deionization (MCDI), and (**c**) flow-electrode capacitive deionization (FCDI) systems.

CDI is one of the most widely studied electrochemical water desalination technologies [11,67,68]. Unlike filtration processes, where the feed and permeate are separated by a semi-permeable membrane (Figure 1a), CDI consists of two electrodes connected by a flow channel, in which an electric circuit is in contact with saline water (Figure 2a) [69]. A CDI system has a cathode and an anode at each end, providing an electrical double layer (EDL) of stored ions dissolved in water after the electrode charge during the first cycle. The second cycle discharges the ions, resulting in concentrated water. Carbon electrodes with good electrical characteristics, low cost, and many fine pores (<2 nm micropores) have been widely used [70]. Early CDI studies focused on improving electrode properties such as salt adsorption capacity and specific capacitance (Table 1). For instance, the use of three-dimensional hierarchical carbon showed the salt adsorption capacity of 26.80 mg g^−1^ [43], which is nearly triple the capacity of the non-treated carbon electrode [34,35]. However, a recent study showed that thermodynamic efficiency could not be proportionally increased, regardless of the electrode properties [71]. Electrode development is relatively insignificant above a certain lever, because overall CDI performance is influenced not only by the electrode performance, but also by other factors, such as spacers, flow patterns, and external resistance [71].

Membrane capacitive deionization (MCDI) with an ion exchange membrane attached to the electrode surface has been widely studied to improve the ion selectivity (or ion storage capacity) of low-performance CDI electrodes (Figure 2b) [69]. MCDI shows greatly enhanced desalination performance by suppressing Faradaic reactions (i.e., chlorine reactions and water splitting) and providing more ion storage sites than the conventional CDI process [72]. Additionally, electrical energy in MCDI operation could be reduced with a combined technique in which a polarity reversal follows a short circuit [73]. These advantages of MCDI imply that the use of ion-exchange membranes in electrochemical processes, such as battery deionization (BDI) and electrodialysis (ED), could help enhance desalination performance over conventional CDI in future research owing to the Donnan potential of the ion-exchange membrane [10,74]. Donnan potential can be caused by imbalances in ion concentration across the ion-exchange membrane. Thus, the use of ion-exchange membranes in an electrochemical system could increase the cell potential without additional force.

Flow-electrode capacitive deionization (FCDI) using suspended electrodes (mostly carbon-based electrodes) has been developed and actively studied to overcome the limited ion adsorption capacity and the need for the desorption cycle (Figure 2c) [72,75]. However, even in FCDI, Faradaic reactions cannot be excluded entirely when the particle electrode materials react outside the cell to adjust the pH of the solution.

Hybrid desalination systems that use sequentially coupled high salt rejection membrane (NF and RO) and CDI-based processes (MCDI and FCDI) are attracting more attention [76,77]. As standalone CDI possesses a relatively low salt removal rate in desalting highly saline water >3000 mg/L [69,76], NF and RO could play an important role in providing feed water quality suitable for CDI. The NF-MCDI system could be more energy efficient than RO for brackish water treatment (≤10,000 mg/L) and meet drinking water standards (≤ 500 mg/L) [69], because NF membranes require lower operating pressure and electrical power consumption compared to RO membranes owing to the lower membrane resistance and concentration polarization.

The energy stored in the EDL during the charging step can theoretically be recovered during the discharging step using a buck–boost converter [78,79]. The energy can briefly be stored in an extra circuit connected to a CDI unit when the potential and current signs are in opposite directions. Thus, to calculate the *TEE* of CDI, the energy recovery *(E_R_*; kWh m^−3^) during the discharge step can be considered as:

(5) $$TEE=\frac{\Delta g}{SEC_{CDI}-E_{R}}\times 100\left(\%\right)$$

where *SEC_CDI_* is the specific energy consumption of constant-current CDI and can be calculated as [80]:

(6) $$SEC_{CDI}=\frac{I\int_{o}^{T_{c}}Vdt}{V_{total}}$$

where *V* is the applied voltage, *I* is the applied current, and *T_c_* is the charging step time. *V_total_* is the total water volume produced during one complete cycle (charging and discharging steps). The symbols *I* and *V* in Equation (6) must be transposed in constant-voltage CDI. Energy recovery can also be considered, because the energy released during the discharging step could be harvested with a converter [78].

The following equation can be used to obtain the theoretical ∆*g* dependence on the extent of desalination in CDI [18].

(7) $$\Delta g=2RT_{a}\left\{\frac{C_{0}}{Y}\ln\left[\frac{C_{0}-YC_{D}}{C_{0}\left(1-Y\right)}\right]-C_{D}\ln\left[\frac{C_{0}-YC_{D}}{C_{D}\left(1-Y\right)}\right]\right\}$$

where *R* is the ideal gas constant (8.314 J K^−1^ mol^−1^), *T_a_* is the absolute temperature (assumed to be 298 K), *C*_0_ is the feed concentration, and *C_D_* is the stabilized product water concentration. The water recovery (*Y*) of CDI is often assumed to be 0.5 when an identical time is used for the charging and discharging steps at the same flow rate. Finally, the TEE for the RO and CDI processes (Equations (3) and (5)) can be used with the productivity of fresh (desalinated water) to compare the energy efficiency of each process.

### 3.2. Battery Deionization

**Figure 3:**
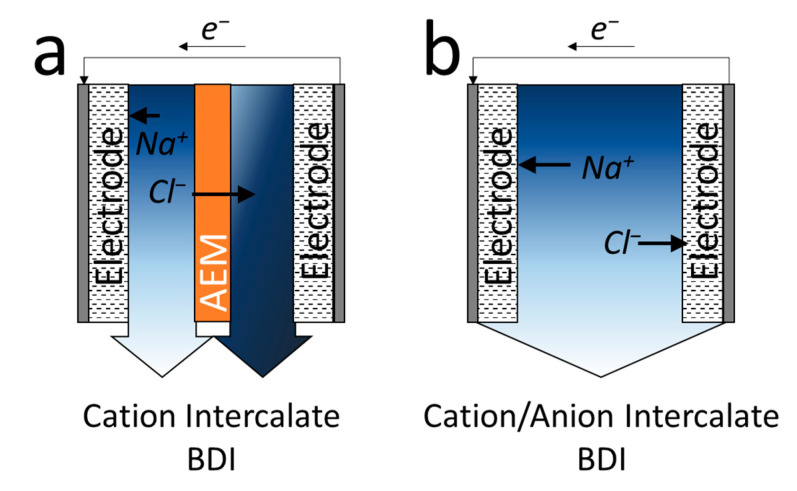
Schematic and working principle of (**a**) cation intercalate battery deionization (BDI) (symmetric) and (**b**) cation/anion (asymmetric) intercalate BDI processes.

To convert Faradaic reactions—which are problematic reactions in the CDI process [72]—to the useful reactions, a Faradaic deionization process was developed to directly exchange the flux of electrons and ions [81]. This is also commonly referred to as the cation intercalation desalination [82,83,84] or BDI process [10,85,86,87] and primarily uses electrodes that can intercalate sodium ions (similar to that of battery electrodes for lithium-ion intercalation) (Figure 3a).

For the symmetric BDI process, sodium ions are intercalated into the cathode during the charging step, and the counter chloride ions migrate through the anion exchange membrane to the opposite stream to maintain the electrical neutrality of the cell. In the second cycle, the cathode becomes the anode, releasing previously adsorbed sodium ions; thus, the desalination stream becomes the concentrate stream (Figure 3a). The reaction mechanism shows that BDI has several inherent advantages over CDI. First, the thermodynamic energy efficiency of BDI could be higher than that of CDI, because BDI directly utilizes Faradaic reactions as redox reactions (reduction and oxidation of electrodes) and can also be operated at a lower voltage (~1.0 V) than conventional CDI (~1.2 V). Additionally, switching water streams allows the production of semi-continuous fresh water (water recovery ratio of ~100%), whereas the CDI process typically has a water recovery ratio of 50%. During the charging step, the cleaned water is generated on the left stream (Figure 3a), whereas the treated water is generated on the right stream during the discharging step in the following operation.

The use of ion exchange membranes in electrochemical processes, such as BDI and MCDI, could render the system more energy (or charge) efficient because of the Donnan potential of the ion exchange membranes [88,89]. Therefore, the electrode development is important; however, the design of the electrochemical processes also plays an important role in the further application of these processes for water desalination. Although cation intercalation materials have been widely studied, the dual-ion intercalation (or asymmetric) system has also been proposed by using both cation and anion intercalation electrodes (Figure 3b) [90,91,92,93]. Unlike typical CDI that utilizes EDL for ion storage, cations and anions can be directly intercalated into the electrode in the BDI system. Thus, the BDI system often showed superior salt adsorption capacity (>50 mg-Na^+^/g [10,87]) compared to that of the CDI system (<30 mg-Na^+^/g [67]) depending on the electrode geometry and the selection of materials.

### 3.3. Electrodialysis

**Figure 4:**
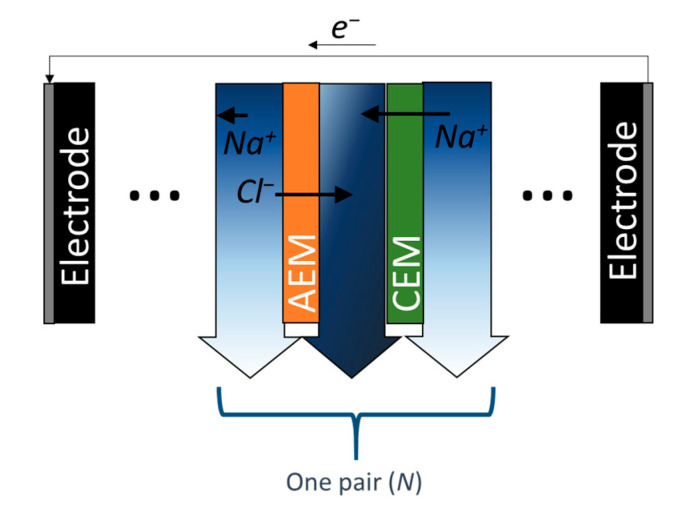
Schematic and working principle of electrodialysis (ED) that becomes the reverse electrodialysis (RED) mode where current flows in the external circuit when solutions with different salinities flow without applying current to the system.

ED is a desalination technique that alternately arranges a pair of cation and anion exchange membranes between two electrodes, a cathode, and an anode (Figure 4) [94]. ED utilizes the migration of ions through ion exchange membranes, whereas CDI uses ion adsorption on the EDL or electrode surface. A recent simulation study showed that ED possibly outperforms CDI because of the diminished influence of Faradaic reactions at the electrodes [74]. Therefore, the use of an ion exchange membrane in the ED system increased the overall energy efficiency of the system, similar to the case of MCDI and BDI. However, the cost of the relatively expensive ion exchange membrane should be considered when designing large-scale systems. The reverse use of ED allows electricity to be produced from two solutions with different chemical potentials through reverse electrodialysis (RED) [94,95]. The working principle of RED is similar to that of the desalination battery using CDI or BDI; electricity is produced with an electrode with (or without) an ion-exchange membrane in between [96,97].

## 4. Energy and Resource Recovery

Membrane (M) and electrochemical (E) processes, such as PRO (M), RED (E), BDI (E), or seawater battery (SWB: E) can be used for salinity gradient energy production as well as water desalination [98,99,100]. However, in the case of PRO, only a limited energy density (<1.5 W m^−2^) can be obtained by using the well-known combination of seawater and river water [65]. Although several pilot studies have been reported, the application of RED is also limited by its low power density of <1.0 W m^−2^ [100]. A remarkably high (>12 W m^−2^) peak power density was reported for BDI; however, the longevity of the electrode should be more carefully investigated in the future [101]. Although rechargeable SWB has been reported, the low cycle performance (~84% over 40 cycles) needs to be improved to compete with commercial lithium batteries [102]. Additionally, research on fouling on the electrode surface for most of the aforementioned systems is insufficient. Thus, obtaining high power density during long-term operation remains a challenge.

Hydrophobic or electrically conducting membranes have been recently developed to efficiently recover (or capture) valuable resources, such as ammonia and phosphate, from wastewater [103,104]. These membranes facilitate the conversion of ammonium into ammonia or enhance the mass transfer of the target compound. The use of electrochemical cells such as CDI, ED, and BDI can efficiently produce nutrients or rare element-enriched streams from wastewater or seawater [85,86,105,106,107,108,109,110], primarily because most of the nutrients in wastewater are present in ionic forms, and the electrochemical process separate (or intercalate) the charged ions using electrostatic (or Faradaic) reactions. For instance, a three-staged BDI system successfully produced an ammonium-enriched stream from 5 mM to 32 mM using synthetic wastewater [85]. However, the cost evaluation for large-scale operation with real (not synthetic) water must be carefully investigated for membrane and electrochemical processes.

## 5. Perspectives for Future Desalination and the Role of Nanotechnology

Membrane processes have seen remarkable growth over the last few decades and their energy consumption of ~4 kWh m^−3^ [3,6] is competitive enough compared to other processes. The RO market is technologically mature enough to satisfy most of the seawater desalination demand (>60%), compared to other technologies [111]. Therefore, it is urgent to continue the research on minimizing environmental impact by developing a cleaning method using less harmful chemicals rather than further reducing the energy consumption of the RO process. Because of the several year-lifespan of TFC RO membranes, longevity needs to be secured for a new class of channel-type or two-dimensional membranes.

Electrochemical processes can easily achieve high energy efficiency by reducing the gap (often <300 μm) between the electrodes. However, this approach could significantly lower the productivity of the process (freshwater production), because the gap between the electrodes proportionally reduces the working volume. Therefore, an optimal effect on the energy consumption per unit of water produced is unlikely. The overall productivity can be maintained by simultaneously increasing the electrode area; however, the capital cost of the system inevitably increases. Therefore, future research should be carefully designed in consideration of both energy efficiency and water productivity. Additionally, the energy consumed by the pretreatment method or feed pump has often been overlooked. The fouling phenomenon (electrode longevity) is another critical issue that needs to be addressed. Fundamental organic and inorganic fouling experiments have been reported for CDI and ED [112,113,114,115,116], but fouling phenomena are not yet fully understood, as electrochemical processes are in the early stages of research compared to membrane processes. Moreover, a limited number of operations with relatively short cycles (mostly <100) have been reported for electrochemical processes [80,85,86,87]. Finally, high-performance electrodes are often challenging to mass-produce because of the use of expensive materials and technical difficulties in synthesis. Comprehensive case studies should focus on the aforementioned parameters to make electrochemical technology commercially successful. After the electrochemical processes overcome these present limitations, the comparative advantage over other technologies must be calculated by considering the TEE and overall productivity of the system.

**Figure 5:**
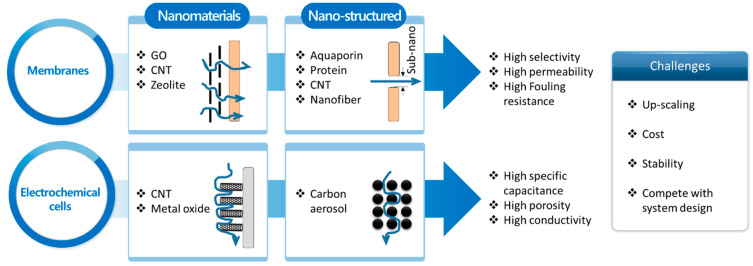
Current role and remaining challenges of nanotechnology for water desalination using membranes and electrochemical cells.

Nanotechnologies, such as nanomaterials [25,28,29,30,117,118] and nano-structure design [27,31,119], have been extensively employed to overcome the current limitations of membranes and electrochemical processes for water desalination (Figure 5). Several materials and techniques have been applied to control the structure or functionality of the material and to produce a highly efficient membrane or electrode. Highly selective (>99.75% salt rejection), permeable (>1.2 L m^−2^ h^−1^ bar^−1^), and fouling-resistant membranes have been developed by applying graphene oxide (or reduced graphene oxide), carbon nanotubes (CNTs), zeolite, aquaporin, protein, and nanofiber [25,27,28,29,30,31]. Superior performance electrodes have been synthesized to have high specific capacitance (>100 F g^−1^), porosity (>50%), and conductivity (>25 S cm^−1^) using CNTs, metal oxides, and carbon aerosols [68,117,118,119]. However, despite continued investigation, the development of high-performance membranes or electrodes using nanotechnology remains challenging. The difficulty of the synthesis for these materials often limits up-scaling at a reasonable cost [120]. For instance, the synthesis of CNTs generally requires chemical vapor deposition using a silicon wafer, which limits the size of the resultant materials smaller than several tens of centimeters [121,122,123,124]. Thus, the employment of only CNTs to fully substitute the active layer (i.e., polyamide layer for RO membrane) is particularly difficult in the industrial scale [123,124]. Therefore, the most promising approach to date could be a hybrid method that applies a small amount of nanomaterials to existing membranes or electrodes [25,125]. In contrast to the direct incorporation of nanomaterial, a nano-structure approach using nanofibers could be employed in a large-scale fabrication as a roll-to-roll synthesis method of nanofiber substrate has been proposed [27]. However, the substrate layer of typical membranes or electrodes is not directly in contact with the feed solution in most processes [25]; hence, the corresponding system should be carefully designed to fully exploit the nano-structures’ functionality. Another challenge is the long-term stability of each material, which has been partially investigated [126,127,128]. Even the widely used carbon material itself, in particular for electrochemical processes, has been often tested in <1000 min (Table 1), which is relatively shorter than industrial needs (often required for several months of operation). As nanomaterials have a large surface area and high reactivity [129,130], their performance decay over time is expected to be significant, implying that the long-term stability might be critical in the practical application of nanomaterials. Moreover, a recent study claimed that the system design could have a greater impact on the overall system performance rather than the effort to develop advanced materials [71]. Therefore, the future use of nanotechnologies in material development should consider these challenges to provide a new pathway for efficient water desalination.

## Figures and Tables

**Table 1:** A summary of enhanced and unexplored performances of the representative polyamide (RO) or carbon (CDI) materials in water desalination. Water permeance and selectivity of commercial thin-film composite (TFC) RO membrane are ~1 LMH/bar at 99% NaCl rejection [32,33]. The salt adsorption capacity of non-treated activated carbon is ~10 mg g^−1^ [34,35].

Process	Type of Membranes (RO) or Electrodes (CDI)	Performances Enhanced	Performances to be Further Explored	Refs.
RO	Multi-layered polyamide	- ~60% flux increase (1.66 ± 0.20 LMH/bar) and higher salt rejection (98%) ^a^ - Better fouling resistance to bovine serum albumin (BSA)	- Fouling experiment with seawater	[23]
	Hydrophilic additives incorporated polyamide	- Flux enhancement (up to 5.77 LMH/bar) ^b^ - Salt rejection of >98.8%	- Fouling resistance	[24]
	Co-solvent induced polyamide	- Twice water flux (2.78 LMH/bar) ^c^ - Salt rejection of 99%	- Long-term stability- Fouling resistance	[22]
	Polyamide synthesized under controlled solution pH	- Flux of > 1.55 LMH/bar ^b^ - Salt rejection of >97%	- Fouling resistance- Chlorine tolerance	[36]
	Polyelectrolyte coated polyamide	- Organic and biofouling control - ~10% flux reduction with a slight increase in salt rejection (>98%) ^d^	- Seawater tests	[37,38]
CDI	Nitrogen-doped porous carbon	- Salt adsorption capacity of 14.91 mg g^−1 e^ - Specific capacitance of 290 F g^−1^	- Long-term stability of >1000 min	[39]
	Nitrogen-doped graphitic porous carbon	- Salt adsorption capacity of 17.73 mg g^−1 f^ - Specific capacitance of 307 F g^−1^		[40]
	Phosphorus-doped 3D carbon nanofiber	- Salt adsorption capacity of 16.20 mg g^−1 e^ - Specific capacitance of 295 F g^−1^		[41]
	Ca-alginate coated-carbon	- Salt adsorption capacity of 14.20 mg g^−1 g^		[42]
	N, P co-doped 3D hierarchical carbon	- Salt adsorption capacity of 26.80 mg g^−1^ ^h^ - Specific capacitance of 221 F g^−1^	- Fouling tests	[43]

Note: LMH/bar: L m^−2^ h^−1^ bar^−1^; mg g^−1^: the amount of salts adsorbed by electrode mass. Testing conditions: ^a^ 500 ppm NaCl at 10 bar; ^b^ 2000 ppm NaCl at 15.5 bar; ^c^ 2000 ppm NaCl at 15 bar; ^d^ 2000 ppm NaCl at 15−41 bar; ^e^ 1000 ppm NaCl at 1.2 V; ^f^ 500 ppm NaCl at 1.4 V; ^g^ 1110 ppm NaCl at 1.2 V; ^h^ 500 ppm NaCl at 1.2 V.
